# Patriline Differences Reveal Genetic Influence on Forewing Size and Shape in a Yellowjacket Wasp (Hymenoptera: Vespidae: *Vespula flavopilosa* Jacobson, 1978)

**DOI:** 10.1371/journal.pone.0130064

**Published:** 2015-07-01

**Authors:** Adrien Perrard, Kevin J. Loope

**Affiliations:** 1 Division of Invertebrate Zoology, American Museum of Natural History, New York, New York, United States of America; 2 Department of Neurobiology and Behavior, Cornell University, Ithaca, New York, United States of America; Universidade de São Paulo, Faculdade de Filosofia Ciências e Letras de Ribeirão Preto, BRAZIL

## Abstract

The wing venation is frequently used as a morphological marker to distinguish biological groups among insects. With geometric morphometrics, minute shape differences can be detected between closely related species or populations, making this technique useful for taxonomy. However, the direct influence of genetic differences on wing morphology has not been explored within colonies of social insects. Here, we show that the father’s genotype has a direct effect on wing morphology in colonies of social wasps. Using geometric morphometrics on the venation pattern, we found significant differences in wing size and shape between patrilines of yellowjackets, taking allometry and measurement error into account. The genetic influence on wing size accounted for a small part of the overall size variation, but venation shape was highly structured by the differences between patrilines. Overall, our results showed a strong genetic influence on wing morphology likely acting at multiple levels of venation pattern development. This confirmed the pertinence of this marker for taxonomic purposes and suggests this phenotype as a potentially useful marker for phylogenies. This also raises doubts about the strength of selective pressures on this phenotype, which highlights the need to understand better the role of wing venation shape in insect flight.

## Introduction

Morphology is the result of complex interactions between genetic and environmental influences on the organism during development [1]. Being the most obvious interface between the organism’s genetic background and the environment, morphological characters have long been studied to classify organisms and to understand their evolution. Among insects, wing venation is one of the most studied structures due to its strong taxonomic signal [2,3], its frequent occurrence in the fossil record [4,5], and its functional importance [6,7]. With the development of geometric morphometric methods, more and more studies use wing venation shape as a morphological marker to distinguish groups, including species of bees and wasps [3,5,8]. Wing venation shapes differ between closely-related species, but also populations, sex and castes within populations [9–12]. Monteiro and collaborators [13] even found significant variation of this marker between colonies of honeybees, suggesting the possibility of intraspecific genetic effects on venation. However, while they inferred an estimate of the wing shape heritability, their data enabled no direct observation of the influence of the genotype on this phenotype.

The wing is known to be influenced by external factors such as temperature, diet and cell size [12,14,15]. Furthermore, wing shape variation tends to decrease as genetic distance decreases across taxa [3]. The relative proportion of the shape variation caused by these external factors is thus expected to increase when genetic variation lowers. The observed differences in wing shapes could thus be caused by environmental variation as much as genetic differences, which could be a problem when taxonomic groups are determined with this marker [16,17]. Genetic effects on insect wing shapes have been detected in *Drosophila* through mutation accumulation [18]. However, these results were restricted to organisms reared in artificial conditions, and recent studies have shown that the genetic process of venation formation is different in Hymenoptera [19]. In order to assess the importance of genetic variation on wing shape in the Hymenoptera, we tested for the first time whether patrilines could be distinguished on the basis of their wing venation.

The social Hymenoptera are particularly useful subjects for the study of the genetic basis of morphology [20–22]. In species like honeybees and yellowjackets, nests contain numerous adults derived from a single mother, the queen. For species with queens that mate with multiple males, identifying full-sibling groups of daughters (patrilines) is straightforward, once the queen’s genotype is known. Furthermore, the father’s genetic influence is identical over its entire patriline because male Hymenoptera are haploids. The existence of multiple genetic lines within the common environment of the yellowjacket nest allows the measurement of the relative roles of genotype and environment in explaining phenotypic variation.

In this paper, we explore the genetic effects on wing size and shape of the yellowjacket wasps *Vespula flavopilosa* (Jacobson, 1978). We first determined paternity for workers from two colonies using microsatellite markers before testing whether wing size and shape differed between these patrilines.

## Material and Methods

### Sampling

The two mature colonies of *V*. *flavopilosa* were collected in Ithaca (NY, USA, 42°27’10.25”N 76°28’36.78”W) in late summer of 2013 (Colony 1: August 23^rd^; Colony 2: September 10^th^). The nests were collected on private land with verbal authorization from the land owner. No specific permits were required for the collection of this abundant, non-endangered species. Colonies were anesthetized overnight using CO2 prior to the collection. Adult workers were then stored at -20°C until DNA was extracted. These colonies were chosen from among 10 colonies, studied in a previous analysis of the species [23], based on the number of available workers and the number of observed patrilines.

### Genetic analysis

We extracted DNA from 116 workers per colony, as well as the mother queen, by placing a single antenna or leg in 100 μl of 10% Chelex solution (Chelex 100, 100–200 mesh, Bio-Rad), then incubating for 20 minutes at 95°C. We refrigerated or froze the supernatant before PCR.

We genotyped each worker at five variable loci (LIST2004, RUFA05, RUFA13, RUFA19, VMA3) using two multiplex PCR reactions [24–26]. Products at each locus were distinguished by employing simple dye-labeled primers (Applied Biosystems) and a 3-primer method [27], as well as fragment size for those labeled with the same dye. Each 10 μl PCR reaction included 1 μl of extracted DNA, 5 μl Qiagen master mix (Qiagen Type-It Microsatellite Kit, Qiagen Inc.), 0.2 μl of each reverse primer, 0.2 μl (dye-labeled) or 0.1 μl (3-primer labeled) of each forward primer, 0.15 μl FAM-labeled 3-primer tag for each 3-primer-labeled primer pair, and water. PCR reaction conditions were 95°C for 15 minutes, 35 cycles of 95°C for 30 seconds, 50°C for 90 seconds, 72°C for 60 seconds, followed by 60°C for 30 minutes. The fragment analysis was performed on an ABI-3730xl sequencer using 0.5 μl PCR product combined with 15 μl HiDi Formamide and 0.15 μl LIZ 500 internal size standard (Applied Biosystems). Allele sizes were called using GeneMarker (SoftGenetics LLC) and checked twice by eye.

### Assigning paternity

We used Colony2 v2.0.4.1 [28] to find the maximum likelihood configuration of paternity assignments for all genotyped workers. Workers that failed to amplify at more than one locus were excluded from the analysis. We included 116 genotyped workers from Colony 1 and 112 genotyped workers from Colony 2, as well as 157 workers from the eight additional colonies reported in the previous study [23]. In the few cases where Colony2 initially assigned a worker to a matriline from a different colony, the anomalous worker genotype was checked against the genotype of the queen from that colony. If she shared an allele with the queen at all loci, a maternal sibship constraint was entered into Colony2 containing all workers in that colony that did not differ from the queen for both alleles at any locus. In all cases, the subsequent run of the likelihood analysis assigned the worker to an additional patriline from that colony.

The probability that two randomly selected males shared the same multi-locus genotype (non-detection error) was assessed using the formula $\prod_{j-1}^{n}\sum_{i-1}^{k}q²ij$, where *q*_*ij*_ is the frequency of the *i*th allele at the *j*th locus [29]. Allele frequencies were calculated and adjusted by Colony2.

### Morphometric measurements

The right forewing of every specimen was dissected and mounted in a water droplet between microscopic slides. In order to obtain repeatable pictures of wings that were as coplanar as possible, we removed the thick part at the base of the wings beforehand. Pictures were taken with a Visionary Digital imaging system, using Infinity lenses and a Canon 60D camera with a constant magnification.

**Fig 1:**
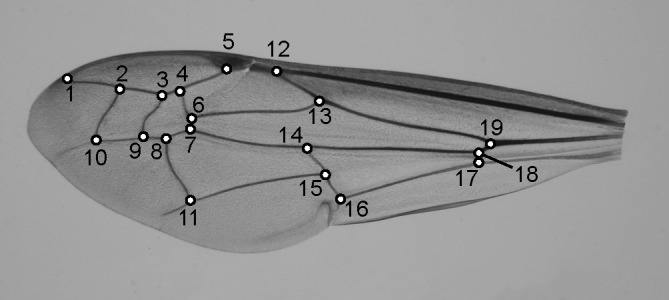
Position of the 19 landmarks used to characterize the size and shape of the forewing.

Forewing size and shapes were assessed using 19 2D landmarks recorded with TPSDig2 software [30] (Fig 1). Landmarks of all specimens were superimposed once, using a generalized Procrustes analysis (GPA; [31]). This analysis extracts information of location, orientation and size from the raw landmark coordinates measured on the pictures in order to retain only the geometric shapes. Aligned landmark coordinates were then projected into the linear tangent space [32]. Shape variables were assessed as the scores of the 34 Principal Components (PC) with eigenvalues greater than zero from a Principal Component Analysis (PCA) on these tangent coordinates. In this multidimensional space, each point represented a potential wing shape and each vector depicted a shape change.

The forewing size was estimated by the log-transformed centroid size from the Procrustes analysis [33].

### Measurement error

As the observed shape variation was very low, we verified that the variation due to measurement error was lower than the biological variation. Measurement error was divided between two potential sources of variation: the preparation of the wings and the accuracy of the digitization. In order to compare the scale of this variation relative to the biological variation, the wing of 39 specimens from Colony 1 were prepared twice and landmarks were digitized twice for each picture. The scale of the measurement errors was assessed by comparing the mean squares of wing shapes variation [34]. Mean squares were computed from a Procrustes Analysis of Variance (Procrustes ANOVA) using hierarchical system of four nested factors: patriline, individual, wing mounting and landmark digitization.

### Analyses

First, the variation in wing shape between colonies and between patrilines was observed on the whole sample. The wing size difference between colonies and between patrilines was tested using an analysis of variance (ANOVA) with two nested factors: colony and patriline. Allometry, the effect of size on shape, and wing shape differences between colonies and between patrilines within colonies were tested using a multiple analysis of covariance (MANCOVA). The actual difference in wing shape between patrilines was further estimated by the rate of correct reassignments using canonical variates with leave-one-out cross-validation. Reassignments were estimated using 2 to 34 PCs because canonical variates may be influenced by the dimensionality [35].

Because several patrilines had low sample sizes, further analyses were restricted to balanced subsamples (34 individuals) drawn from the two main patrilines in each colony. Wing size differences between these patrilines were tested in each colony using Student’s t-tests. The influence of patrilines on wing shape was tested on both shape data and allometry-corrected data using the two-sample Hotelling’s T² test. Allometry-corrected data were assessed as the residuals of colony shape data from the common allometric component. The common allometric component was estimated for each colony by a multivariate regression of pooled patriline shape variables on size. Shape differences between colonies and between the main pair of patrilines of each colony were also compared in terms of angles between the vectors within the tangent space. If the differences between these three groups were similar, the three vectors describing these shape changes should have a similar direction. We thus tested whether the angles between these vectors of group differences were significantly lower than angles between pairs of shapes randomly drawn from the sample.

Morphometric analyses were performed with the software R and the packages MASS, Rmorph and geomorph [36–39].

## Results

We detected four patrilines in each colony. Non-detection error was low (1.5%), suggesting a low probability of mistakenly identifying workers from two distinct patrilines as the offspring of a single male. Patriline frequencies varied from 14.66% to 37.07% in Colony 1 and from 5.36% to 42.86% in Colony 2. Out of the 228 successfully genotyped specimens, eight had the wing too damaged for proper measurements. A total of 220 wings were thus measured for size and shape (Table 1).

**Table 1:** Number of individuals sampled from each patriline.

Patriline	Colony 1	Colony 2
1	43	44
2	34	41
3	22	12
4	17	6

### Differences in wing sizes

Workers presented significantly different wing sizes between the two colonies (F = 17.525, df = 1, P = 0.00004) as well as between patrilines within each colony for the whole sample (F = 4.081, df = 6, P = 0.00068). Wing sizes were significantly different between the two main patrilines of Colony 2 (Student’s t-test: t = 2.7933, df = 65.813, P = 0.00683). However, the two main patrilines of Colony 1 presented non-significant size differences (Student’s t-test: t = 0.44, df = 65.976, P = 0.6614). Furthermore, the size variation between colonies and between patrilines was much lower than the size variation observed within the patrilines (S²_inter-colony_ = 2.54×10^-4^, S²_inter-patrilines_ = 4.04×10^-4^, S²_intra-patriline_ = 15.42×10^-4^).

### Shape variation

Measurement error was more than a hundred times lower than the differences in wing shape between patrilines (Table 2). The variation between individuals within a patriline was five times higher than the error induced by the wing preparation, and more than 292 times higher than the error related to landmark digitization. These results confirmed that the observed biological variation of the wing shape could not be explained by measurement error.

**Table 2:** Measurement error based on 39 specimens from 4 patrilines of Colony 1.

Effect	Sum of Squares	Df	Mean Squares (10^-5^)
Patriline	0.00624	102	6.11910
Individual	0.01309	5168	0.25344
Wing mounting	0.00278	5304	0.05242
Landmark digitization	0.00009	10608	0.00087

Sum of squares and degrees of freedom are based on a Procrustes ANOVA. Df: Degrees of freedom.

**Fig 2:**
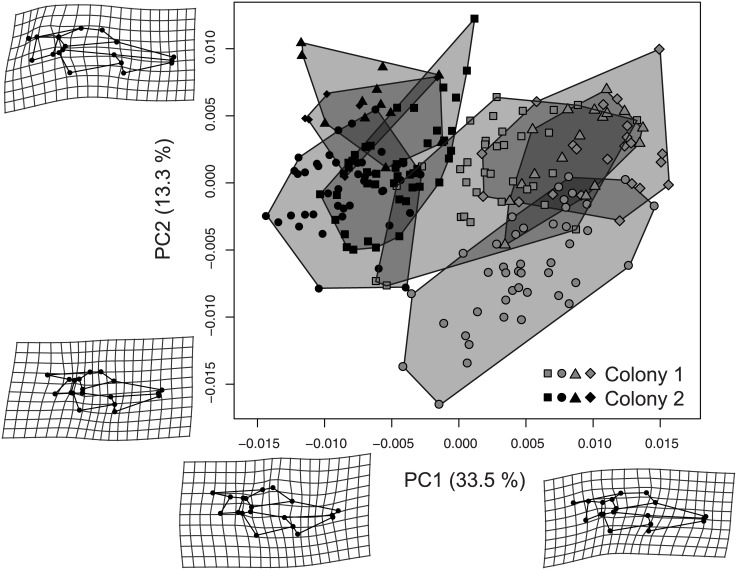
Two first Principal Components (PC) of the shape variation of the wing venation patterns. The region occupied by each patriline was indicated in grey. The shape changes described by each PC were amplified threefold to permit visualisation. Percentages of the axes indicate the proportion of the total shape variation explained by each PC. The apparent overlap of colonies and patrilines on this representation was mostly due to the projection of the 34-dimensions shape space on the plane of the 2 first PCs: canonical variate analyses with cross-validation showed that the overlap between patrilines was much lower when taking more dimensions into account. Squares: 1^st^ patriline of each colony; circles: 2^nd^ patrilines; triangles: 3^rd^ patrilines; diamonds: 4^th^ patrilines.

The first two principal components (PCs) of the shape variation of the whole sample separated the two colonies and revealed a partial structure of the different patrilines within colonies (Fig 2). These PCs accounted for 33.5% and 13.3% of the total shape variation respectively. Additional differences between pairs of patrilines were observed on several of the other PCs. The MANCOVA showed that allometry—the influence of size on the wing shape—was significant, as were the shape differences between colonies and among patrilines (Table 3). The allometry was significantly different between colonies, but not among patrilines within colonies. The average procrustes distance between patrilines’ mean shapes was only slightly higher than the average procrustes distance between an individual shape and the mean shape of its patriline (0.01422 ± 0.00399 between patriline mean shapes; 0.00951 ± 0.00212 within patrilines). However, the canonical variates confirmed that different patrilines had different wing shapes: the rate of individuals that could be attributed to their correct patriline on the basis of their wing shape was high. When using cross-validation, it ranged from 65% when using 2 PCs—46.6% of the total shape variation—to 99.09% with 19 PCs—96.53% of the total variation (S1 File). The wing shapes of different patrilines thus occupied overlapping, but largely distinct, regions of the shape space.

**Table 3:** Effect of wing size, colony and patrilines on the wing shapes of the yellowjackets.

Effect	Df	Pillai	approx F	num Df	den Df	P
Size	1	0.8135	21.943	34	171	0
Colony	1	0.9272	64.031	34	171	0
Patriline	6	4.0490	10.743	204	1056	0
Size * Colony	1	0.2750	1.908	34	171	0.00394
Size * Patriline	6	1.0632	1.115	204	1056	0.14897
Residuals	204					

Df = Degree of freedom; Pillai = Test value; approx F = Multivariate approximation of the F-statistic value; num Df / den Df = Degrees of freedom of the numerator / denominator; P = Probability.

**Fig 3:**
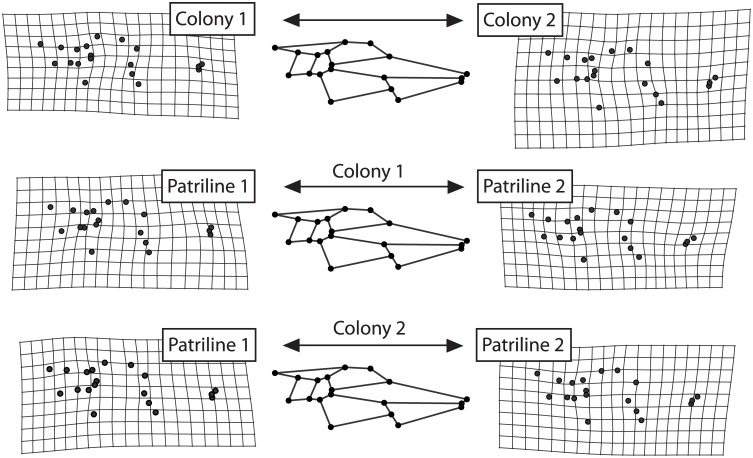
Shape changes between the colony and main patrilines. The shapes depict the difference between the two colonies, the two main patrilines of Colony 1 and the two main patrilines of Colony 2 respectively. The transformation grids show that the changes occur in different regions of the wings and with different combinations of local changes. Due to the small shape variation, shape changes were amplified tenfold.

Within each colony, the two main patrilines were also readily discriminated on the first PCs of colony-based PCAs. The Hotelling T² tests confirmed that the wing shapes of individuals from different patrilines were significantly different (Colony 1, T² = 25.545, df_num_ = 34, df_den_ = 33, P = 0; Colony 2, T² = 17.108, df_num_ = 34, df_den_ = 33, P = 0). Correction for allometry was not required for Colony 1 as the wing size difference between the main patrilines was not significant. Allometry-corrected data confirmed that the difference in wing shape between the two main patrilines of Colony 2 was not due to their size difference (Colony 2, T² = 18.324, df_num_ = 34, df_den_ = 33, P = 0). These differences between colonies and between main patrilines reflected completely different shape changes (Fig 3): the angles between the vectors of these differences were not significantly similar when compared to 10,000 random vectors (θ_ΔCΔP1_: 73.69°, P = 0.2096; θ_ΔCΔP2_: 74.37°, P = 0.2195; θ_ΔP1ΔP2_: 77.76°, P = 0.2710).

## Discussion

Our results clearly show an influence of paternity—and thus of genotype—on the wing morphology of yellowjackets.

In both colonies, genetic analysis revealed four different patrilines, which is congruent with previous studies on paternity in other species of *Vespula* yellowjackets [40] and typical of *V*. *flavopilosa* [23]. We found that the different patrilines presented small but significant differences in wing sizes. Whether this size difference is due to genetic effects or to different rearing conditions cannot be assessed yet. The nest environment of social insects, including social wasps, is remarkably homeostatic [41, 42]. However, we know that the average size of cells, and thus of workers, tends to increase during the development of the colony [43]. The first worker cells built in the colony are smaller than the cells built later in the season, and the use of small cells diminishes with time in favor of larger cells. It would be interesting to assess whether patriline wing size variation may be linked to a change in sperm use patterns through time: some patrilines reared early would develop mostly in small cells while others would benefit from larger cells later. Against this environmental explanation, two studies of sperm use in yellowjackets suggest that patriline representation is constant over the course of colony development [44, 45]. Regardless, the patriline influence on wing size was minimal compared to the size variation found between full-sib individuals. This confirms the results of Kovacs and collaborators [21] who found that the variance explained by patrilines was low and often non-significant in measurements on *V*. *maculifrons* workers. As workers only indirectly affect the colony fitness, worker size may be under less rigidly controlled genetic influence than gyne size [22].

However, our results demonstrated a clear influence of paternity on workers’ wing shapes. Factors such as diet or temperature may affect the wing shape [14,15] and could vary slightly within the nest. Nevertheless, there is no evidence that individuals from different patrilines experienced different developmental conditions. These conditions would likely have an influence on the size of individuals as well [14], but the two main patrilines of Colony 1 presented no size difference. Their difference in wing shape was greater than the one between the two main patrilines of Colony 2, which differed in size as well as shape. Furthermore, even in this second colony, the differences in size between patrilines could only explain a small fraction of these shape differences. These results suggest that the father’s genotype does affect the offspring’s wing shapes, even if environmental factors may have contributed to the wing variation between patrilines. Unfortunately, our sample of two colonies did not allow us to calculate formal population measures such as heritability to quantify the proportions of variation due to genetic and environmental effects. On the other hand, the distinction of the two main patrilines on the first PCs within each colony suggested that genetic difference is the main cause of wing shape variation, at least within these two colonies.

The development of the wing veins has been extensively studied in *Drosophila* (*e*.*g*. [46–48]). These studies found that morphogens such as the Decapentaplegic protein (Dpp) induce the differentiation of the wing imaginal tissue into sclerotized veins. In *Drosophila*, the venation pattern was thought to be defined by the regulated activity of genes coding for these morphogens, such as *dpp* [47]. However, a recent study in Sawflies revealed that pre-patterning components directing Dpp transport were more important in defining the vein locations than the local activity of *dpp* [49]. How this pre-pattern is defined and under which control is still unknown [19]. Nonetheless, our results highlighted that different genotypes from a same population produced structured differences in the venation pattern: the wing venation pattern seemed under a clear genetic influence. The different patrilines were also well discriminated despite the approximately equal magnitude of shape variation between patrilines and within patrilines. This discrimination suggests that the variation within patrilines, likely induced by external factors, occurs in different directions of the shape space than the variation between patrilines, reflecting genetic effects. Finally, these differences were not equivalent, depending on the compared patrilines. Different developmental pathways may have been involved in producing the shape variation between patrilines, suggesting that the observed shape variation likely resulted from mutations across a set of genes involved in regulatory processes of the spatial activity of morphogens like Dpp [19].

Wing venation is often used for insect phylogenies based on morphology (*e*.*g*. [50]), but the venation shape assessed by geometric morphometrics is currently rarely used in this context. This could be due to methodological difficulties [51,52], though new methods are being developed to face this challenge [53,54]. Our results showed a direct link between the wing venation shape and the genotype. This link was detected despite a low genetic variation, suggesting large genetic effects. A strong genetic influence, in combination with variation in multiple independent directions, which lowers potential convergences [55,3], highlight the potential of wing shape as a phylogenetic character. Although the venation shape variation cannot be entirely attributed to the genotype [52,56], its use in phylogenetic analyses could provide additional data for species for which no genetic data are available, including fossil insects [5].

The insect wing is subject to various functional constraints concerning its size, shape and rigidity [57–60]. Consequently, the venation supporting the wing membrane likely undergoes selection on its ability to protect the wing and to control its flexibility during flight [6,55,61]. However, our results show that even small genetic differences within a population induce structured variation of the wing venation pattern. This high sensitivity of the phenotype to genetic differences suggests that the shape of wing venation may not be highly constrained by selection on the wing function: small changes in venation may not alter the flight performances of wasp workers. Further studies would be required to identify the actual influence of the wing venation shape on flight performance, and to compare the evolution of this phenotype to other features of the wing in order to detect whether they evolve under different selection regimes.

## Supporting Information

S1 File Classification tables of the specimens on the basis of their wing shape using Canonical Variates Analyses with leave-one-out cross-validation. The 220 specimens of *Vespula flavopilosa* studied came from two colonies with 4 patrilines each. Each table presents the results when using an increasing number of Principal Components to describe the wing shape (from 2 to 34).
(DOC)

## References

[1] Debat V, David P. Mapping phenotypes: canalization, plasticity and developmental stability. Trends Ecol Evol. 2001;16: 555–561.

[2] Béthoux O. Groundplan, nomenclature, homology, phylogeny, and the question of the insect wing venation pattern. Alavesia. 2008;2: 219–232.

[3] Perrard A, Baylac M, Carpenter JM, Villemant C. Evolution of wing shape in hornets: why is the wing venation efficient for species identification? J Evol Biol. 2014;27: 2665–2675. 10.1111/jeb.12523 25345804

[4] Nel A, Prokop J, Nel P, Grandcolas P, Huang D-Y, Roques P, et al Traits and evolution of wing venation pattern in paraneopteran insects. Journal of Morphology. 2012;273: 480–506. 10.1002/jmor.11036 22162020

[5] Dehon M, Michez D, Nel A, Engel MS, De Meulemeester T. Wing Shape of Four New Bee Fossils (Hymenoptera: Anthophila) Provides Insights to Bee Evolution. PLoS One. 2014;9: e108865 10.1371/journal.pone.0108865 25354170 PMC4212905

[6] Combes SA, Daniel TL. Flexural stiffness in insect wings I. Scaling and the influence of wing venation. J Exp Biol. 2003;206: 2979–2987. 12878666 10.1242/jeb.00523

[7] Klingenberg CP, Debat V, Roff DA. Quantitative genetics of shape in cricket wings: developmental integration in a functional structure. Evolution. 2010;64: 2935–2951. 10.1111/j.1558-5646.2010.01030.x 20482613

[8] Tofilski A. DrawWing, a program for numerical description of insect wings. J Ins Sci. 2004;4: 17–21. 10.1093/jis/4.1.17 PMC528877 15861233

[9] Camara M, Caro-Riaño H, Ravel S, Dujardin J, Hervouet J, De MeEüs T, et al Genetic and morphometric evidence for population isolation of *Glossina palpalis gambiensis* (Diptera: Glossinidae) on the Loos islands, Guinea. J Med Entomol. 2006;43: 853–860. 17017219 10.1603/0022-2585(2006)43[853:gamefp]2.0.co;2

[10] Henry A, Thongsripong P, Fonseca-Gonzalez I, Jaramillo-Ocampo N, Dujardin J-P. Wing shape of dengue vectors from around the world. Infect Genet Evol. 2010;10: 207–214. 10.1016/j.meegid.2009.12.001 20026429

[11] Pretorius E. Using geometric morphometrics to investigate wing dimorphism in males and females of Hymenoptera–a case study based on the genus *Tachysphex* Kohl (Hymenoptera: Sphecidae: Larrinae). Aust J Entomol. 2005;44: 113–121.

[12] Perrard A, Villemant C, Carpenter JM, Baylac M. Differences in caste dimorphism among three hornet species (Hymenoptera: Vespidae): forewing size, shape and allometry. J Evol Biol. 2012;25: 1389–1398. 10.1111/j.1420-9101.2012.02527.x 22551305

[13] Monteiro LR, Diniz-Filho JAF, Reis SF, Araújo ED. Geometric estimates of heritability in biological shape. Evolution. 2002;56: 563–572. 11989686 10.1554/0014-3820(2002)056[0563:GEOHIB]2.0.CO;2

[14] Debat V, Bégin M, Legout H, David JR. Allometric and Nonallometric components of *Drosophila* wing shape respond differently to developmental temperature. Evolution. 2003;57: 2773–2784. 14761056 10.1111/j.0014-3820.2003.tb01519.x

[15] Shingleton AW, Das J, Vinicius L, Stern DL. The temporal requirements for insulin signaling during development in *Drosophila* . PLoS Bio. 2005;3: e289 16086608 10.1371/journal.pbio.0030289 PMC1184592

[16] Baylac M, Villemant C, Simbolotti G. Combining geometric morphometrics with pattern recognition for the investigation of species complexes. Biol J Linn Soc Lond. 2003;80: 89–98.

[17] Villemant C, Simbolotti G, Kenis M. Discrimination of *Eubazus* (Hymenoptera, Braconidae) sibling species using geometric morphometrics analysis of wing venation. Syst Entomol. 2007;32: 625–634.

[18] Houle D, Fierst J. Properties of spontaneous mutational variance and covariance for wing size and shape in *Drosophila melanogaster* . Evolution. 2013;67: 1116–1130. 10.1111/j.1558-5646.2012.01838.x 23550760

[19] Shimmi O, Matsuda S, Hatakeyama M. Insights into the molecular mechanisms underlying diversified wing venation among insects. Proc R Soc Lond B Biol Sci. 2014;281: 20140264. 10.1098/rspb.2014.0264 PMC4100500 25009057

[20] Fjerdingstad EJ. Control of body size of *Lasius niger* ant sexuals—worker interests, genes and environment. Mol Ecol. 2005;14: 3123–3132. 16101778 10.1111/j.1365-294X.2005.02648.x

[21] Kovacs JL, Hoffman EA, Marriner SM, Rekau JA, Goodisman MAD. Environmental and genetic influences on queen and worker body size in the social wasp *Vespula maculifrons* . Ins Soc. 2010;57: 53–65.

[22] Kovacs JL, Goodisman MAD. Effects of size, shape, genotype, and mating status on queen overwintering survival in the social wasp *Vespula maculifrons* . Environ Entomol. 2012;41: 1612–1620. 10.1603/EN12023 23321110

[23] Loope KJ, Chien C, Juhl M. Colony size is linked to paternity frequency and paternity skew in yellowjacket wasps and hornets. BMC Evol Biol. 2014;14: 277 10.1186/s12862-014-0277-x 25547876 PMC4298054

[24] Thoren PA, Paxton RJ, Estoup A. Unusually high frequency of (CT) n and (GT) n microsatellite loci in a yellowjacket wasp, *Vespula rufa* (L.)(Hymenoptera: Vespidae). Insect Mol Biol. 1995;4: 141–148. 8589840 10.1111/j.1365-2583.1995.tb00019.x

[25] Daly D, Archer ME, Watts PC, Speed MP, Hughes MR, Barker FS, et al Polymorphic microsatellite loci for eusocial wasps (Hymenoptera: Vespidae). Mol Ecol Notes. 2002;2: 273–275.

[26] Hasegawa E, Takahashi J. Microsatellite loci for genetic research in the hornet *Vespa mandarinia* and related species. Mol Ecol Notes. 2002;2: 306–308.

[27] Schuelke M. An economic method for the fluorescent labeling of PCR fragments. Nat Biotechnol. 2000;18: 233–234. 10657137 10.1038/72708

[28] Jones OR, Wang J. COLONY: a program for parentage and sibship inference from multilocus genotype data. Mol Ecol Resour. 2010;10: 551–555. 10.1111/j.1755-0998.2009.02787.x 21565056

[29] Boomsma JJ, Ratnieks FL. Paternity in eusocial Hymenoptera. Philos Trans R Soc Lond B Biol Sci. 1996;351: 947–975.

[30] Rohlf FJ. tpsDig v2.16. Department of Ecology and Evolution, State Univ of New York, Stony Brook, NY: Distributed by author.

[31] Dryden IL, Mardia KV. Statistical shape analysis. Wiley Chichester; 1998.

[32] Rohlf FJ. Shape statistics: Procrustes superimpositions and tangent spaces. J Class. 1999;16: 197–223.

[33] Bookstein FL. Morphometric tools for landmark data. New York: Cambridge University Press; 1991.

[34] Zelditch ML, Swiderski DL, Sheets HD. Geometric morphometrics for biologists: a primer. Academic Press; 2012.

[35] Mitteroecker P, Bookstein F. Linear discrimination, ordination, and the visualization of selection gradients in modern morphometrics. Evol Biol. 2011;38: 100–114.

[36] R-Development Core Team. R: A language and environment for statistical computing. Vienna, Austria;

[37] Venables WN, Ripley BD. Modern applied statistics with S. Springer Science & Business Media; 2002.

[38] Adams DC, Otárola-Castillo E. geomorph: an R package for the collection and analysis of geometric morphometric shape data. Methods Ecol Evol. 2013;4: 393–399.

[39] Baylac M. Rmorph: an R geometric and multivariate morphometrics library. Available from the author: baylac@mnhn.fr. 2012;

[40] Foster KR, Ratnieks FL. Paternity, reproduction and conflict in vespine wasps: a model system for testing kin selection predictions. Behav Ecol Sociobiol. 2001;50: 1–8.

[41] Wilson EO. The insect societies. Cambridge: Harvard University Press; 1971.

[42] Jones JC, Oldroyd BP. Nest thermoregulation in social insects. Adv Ins Physiol. 2006;33: 153–191.

[43] Spradbery JP. Wasps An account of the biology and natural history of social and solitary wasps, with particular reference to those of the British Isles. Sidgwick & Jackson Ltd; 1973.

[44] Ross KG. Kin selection and the problem of sperm utilization in social insects. Nature. 1986;323: 798–800.

[45] Goodisman MA, Kovacs JL, Hoffman EA. Lack of conflict during queen production in the social wasp *Vespula maculifrons* . Mol Ecol. 2007;16: 2589–2595. 17561915 10.1111/j.1365-294X.2007.03316.x

[46] De Celis JF, Diaz-Benjumea FJ. Developmental basis for vein pattern variations in insect wings. Int J Dev Biol. 2003;47: 653–664. 14756341

[47] Crozatier M, Glise B, Vincent A. Patterns in evolution: veins of the *Drosophila* wing. Trends Genet. 2004;20: 498–505. 15363904 10.1016/j.tig.2004.07.013

[48] Blair SS. Wing vein patterning in Drosophila and the analysis of intercellular signaling. Annu Rev Cell Dev Biol. 2007;23: 293–319. 17506700 10.1146/annurev.cellbio.23.090506.123606

[49] Matsuda S, Yoshiyama N, Künnapuu-Vulli J, Hatakeyama M, Shimmi O. Dpp/BMP transport mechanism is required for wing venation in the sawfly *Athalia rosae* . Insect Biochem Mol Biol. 2013;43: 466–473. 10.1016/j.ibmb.2013.02.008 23499566

[50] Sharkey MJ, Roy A. Phylogeny of the Hymenoptera: a reanalysis of the Ronquist et al.(1999) reanalysis, emphasizing wing venation and apocritan relationships. Zool Scr. 2002;31: 57–66.

[51] Adams DC, Cardini A, Monteiro LR, O’Higgins P, Rohlf FJ. Morphometrics and phylogenetics: principal components of shape from cranial modules are neither appropriate nor effective cladistic characters. J Human Evol. 2011;60: 240–243. 10.1016/j.jhevol.2010.02.003 20303142

[52] Klingenberg CP, Gidaszewski NA. Testing and quantifying phylogenetic signals and homoplasy in morphometric data. Sys Biol. 2010;59: 245–261. 10.1093/sysbio/syp106 20525633

[53] Catalano SA, Goloboff PA, Giannini NP. Phylogenetic morphometrics (I): the use of landmark data in a phylogenetic framework. Cladistics. 2010;26: 539–549. 10.1111/j.1096-0031.2010.00302.x 34875764

[54] Catalano SA, Goloboff PA. Simultaneously mapping and superimposing landmark configurations with parsimony as optimality criterion. Sys Biol. 2012;61: 392–400. 10.1093/sysbio/syr119 22213710

[55] Mezey JG, Houle D. The dimensionality of genetic variation for wing shape in *Drosophila melanogaster* . Evolution. 2005;59: 1027–1038. 16136802

[56] García Z, Sarmiento CE. Relationship between body size and flying-related structures in Neotropical social wasps (Polistinae, Vespidae, Hymenoptera). Zoomorphology. 2012;131: 25–35.

[57] Outomuro D, Johansson F. Bird predation selects for wing shape and coloration in a damselfly. J Evol Biol. 2015; 10.1111/jeb.12605 25693863

[58] Weber KE. Selection on wing allometry in *Drosophila melanogaster* . Genetics. 1990;126: 975–989. 2127580 10.1093/genetics/126.4.975 PMC1204293

[59] Young J, Walker SM, Bomphrey RJ, Taylor GK, Thomas AL. Details of insect wing design and deformation enhance aerodynamic function and flight efficiency. Science. 2009;325: 1549–1552. 10.1126/science.1175928 19762645

[60] Dirks J-H, Taylor D. Veins improve fracture toughness of insect wings. PLoS One. 2012;7: e43411 10.1371/journal.pone.0043411 22927966 PMC3425546

[61] Mountcastle AM, Combes SA. Wing flexibility enhances load-lifting capacity in bumblebees. Proc Roy Soc B. 2013;280: 20130531 10.1098/rspb.2013.0531 23536604 PMC3619524

