# Supplementary material for: Patriline Differences Reveal Genetic Influence on Forewing Size and Shape in a Yellowjacket Wasp (Hymenoptera: Vespidae: Vespula flavopilosa Jacobson, 1978)
Source: PLoS One. 2015 Jul 1;10(7):e0130064. doi: 10.1371/journal.pone.0130064 (PMC4488467; doi:10.1371/journal.pone.0130064)
Supplement: S1 File — The 220 specimens of Vespula flavopilosa studied came from two colonies with 4 patrilines each. Each table presents the results when using an increasing number of Principal Components to describe the wing shape (from 2 to 34). (DOC) [file pone.0130064.s001.doc]

Perrard & Loope 2015

Supporting information, S1 File:

Classification tables of the specimens on the basis of their wing shape using Canonical Variates Analyses (CVA) with leave-one-out cross-validation. The 220 specimens of *Vespula flavopilosa* studied came from two colonies with 4 patrilines each. Each table presents the results when using an increasing number of Principal Components to describe the wing shape (from 2 to 34). Actual patrilines are in lines, results of the CVA are in columns. Percentage of the total shape variation explained by the principal components is indicated into brackets.

- 2 Principal Components (46.85% of the shape variation);

65% of specimens attributed to their right patriline:

|  |  | Colony 2 | | | | Colony 1 | | | |
| --- | --- | --- | --- | --- | --- | --- | --- | --- | --- |
|  | Patriline | I | II | III | IV | I | II | III | IV |
| Colony 2 | I | 31 | 13 | 1 | - | - | - | - | - |
|  | II | 10 | 29 | 1 | - | 1 | - | - | - |
|  | III | 5 | - | 5 | 2 | - | - | - | - |
|  | IV | 1 | 1 | 4 | - | - | - | - | - |
| Colony 1 | I | - | - | - | - | 38 | - | 5 | - |
|  | II | 5 | - | - | - | 2 | 24 | 1 | 2 |
|  | III | - | - | - | - | 1 | 4 | 15 | 2 |
|  | IV | - | - | - | - | 1 | 5 | 10 | 1 |

- 3 Principal Components (56.12% of the shape variation);

67.73% of specimens attributed to their right patriline:

|  |  | Colony 2 | | | | Colony 1 | | | |
| --- | --- | --- | --- | --- | --- | --- | --- | --- | --- |
|  | Patriline | I | II | III | IV | I | II | III | IV |
| Colony 2 | I | 29 | 13 | 1 | 1 | - | 1 | - | - |
|  | II | 11 | 26 | 1 | 2 | 1 | - | - | - |
|  | III | 4 | - | 7 | 1 | - | - | - | - |
|  | IV | 1 | 1 | - | 4 | - | - | - | - |
| Colony 1 | I | - | - | - | - | 38 | 1 | 1 | 3 |
|  | II | 3 | - | - | - | 1 | 25 | 3 | 2 |
|  | III | - | - | - | - | - | 4 | 15 | 3 |
|  | IV | - | - | - | - | 3 | 4 | 5 | 5 |

- 4 Principal Components (62.41% of the shape variation);

80.45% of specimens attributed to their right patriline:

|  |  | Colony 2 | | | | Colony 1 | | | |
| --- | --- | --- | --- | --- | --- | --- | --- | --- | --- |
|  | Patriline | I | II | III | IV | I | II | III | IV |
| Colony 2 | I | 37 | 5 | 1 | 1 | - | 1 | - | - |
|  | II | 6 | 34 | - | - | - | 1 | - | - |
|  | III | 3 | 1 | 8 | - | - | - | - | - |
|  | IV | 2 | - | - | 4 | - | - | - | - |
| Colony 1 | I | - | - | - | - | 39 | - | 2 | 2 |
|  | II | 2 | 1 | - | - | 1 | 28 | 2 | - |
|  | III | - | - | - | - | - | 3 | 17 | 2 |
|  | IV | - | - | - | - | 3 | 1 | 3 | 10 |

- 5 Principal Components (67.92% of the shape variation);

80.91% of specimens attributed to their right patriline:

|  |  | Colony 2 | | | | Colony 1 | | | |
| --- | --- | --- | --- | --- | --- | --- | --- | --- | --- |
|  | Patriline | I | II | III | IV | I | II | III | IV |
| Colony 2 | I | 40 | 3 | 1 | 1 | - | - | - | - |
|  | II | 6 | 35 | - | - | - | - | - | - |
|  | III | 3 | 1 | 8 | - | - | - | - | - |
|  | IV | 2 | - | - | 4 | - | - | - | - |
| Colony 1 | I | - | - | - | - | 37 | 1 | 2 | 3 |
|  | II | - | 3 | - | - | 1 | 28 | 2 | - |
|  | III | - | - | - | - | - | 3 | 17 | 2 |
|  | IV | - | - | - | - | 3 | 1 | 4 | 9 |

- 6 Principal Components (72.21% of the shape variation);

85% of specimens attributed to their right patriline:

|  |  | Colony 2 | | | | Colony 1 | | | |
| --- | --- | --- | --- | --- | --- | --- | --- | --- | --- |
|  | Patriline | I | II | III | IV | I | II | III | IV |
| Colony 2 | I | 38 | 3 | 3 | 1 | - | - | - | - |
|  | II | 1 | 40 | - | - | - | - | - | - |
|  | III | 3 | 1 | 8 | - | - | - | - | - |
|  | IV | 1 | - | 1 | 4 | - | - | - | - |
| Colony 1 | I | - | - | - | - | 39 | - | 1 | 3 |
|  | II | 1 | 1 | - | - | 2 | 28 | 2 | - |
|  | III | - | - | - | - | 1 | 3 | 18 | - |
|  | IV | - | - | - | - | 3 | - | 2 | 12 |

- 7 Principal Components (76.31% of the shape variation);

87.27% of specimens attributed to their right patriline:

|  |  | Colony 2 | | | | Colony 1 | | | |
| --- | --- | --- | --- | --- | --- | --- | --- | --- | --- |
|  | Patriline | I | II | III | IV | I | II | III | IV |
| Colony 2 | I | 40 | 3 | 2 | - | - | - | - | - |
|  | II | 3 | 38 | - | - | - | - | - | - |
|  | III | 2 | 1 | 9 | - | - | - | - | - |
|  | IV | 2 | - | - | 4 | - | - | - | - |
| Colony 1 | I | - | - | - | - | 39 | - | 2 | 2 |
|  | II | 2 | - | - | - | 1 | 29 | 2 | - |
|  | III | - | - | - | - | - | 2 | 20 | - |
|  | IV | - | - | - | - | 2 | - | 2 | 13 |

- 8 Principal Components (79.30% of the shape variation);

90.45% of specimens attributed to their right patriline:

|  |  | Colony 2 | | | | Colony 1 | | | |
| --- | --- | --- | --- | --- | --- | --- | --- | --- | --- |
|  | Patriline | I | II | III | IV | I | II | III | IV |
| Colony 2 | I | 42 | 1 | 2 | - | - | - | - | - |
|  | II | 1 | 40 | - | - | - | - | - | - |
|  | III | 4 | 1 | 7 | - | - | - | - | - |
|  | IV | - | - | - | 6 | - | - | - | - |
| Colony 1 | I | - | - | - | - | 40 | - | 1 | 2 |
|  | II | 2 | - | - | - | 1 | 31 | - | - |
|  | III | - | - | - | - | 1 | 2 | 19 | - |
|  | IV | - | - | - | - | 1 | - | 2 | 14 |

-9 Principal Components (82.19% of the shape variation);

91.36% of specimens attributed to their right patriline:

|  |  | Colony 2 | | | | Colony 1 | | | |
| --- | --- | --- | --- | --- | --- | --- | --- | --- | --- |
|  | Patriline | I | II | III | IV | I | II | III | IV |
| Colony 2 | I | 42 | - | 3 | - | - | - | - | - |
|  | II | - | 41 | - | - | - | - | - | - |
|  | III | 4 | 1 | 7 | - | - | - | - | - |
|  | IV | - | - | - | 6 | - | - | - | - |
| Colony 1 | I | - | - | - | - | 41 | - | 1 | 1 |
|  | II | 2 | - | - | - | 1 | 31 | - | - |
|  | III | - | - | - | - | 1 | 2 | 19 | - |
|  | IV | - | - | - | - | 1 | - | 2 | 14 |

- 10 Principal Components (84.85% of the shape variation);

93.64% of specimens attributed to their right patriline:

|  |  | Colony 2 | | | | Colony 1 | | | |
| --- | --- | --- | --- | --- | --- | --- | --- | --- | --- |
|  | Patriline | I | II | III | IV | I | II | III | IV |
| Colony 2 | I | 43 | - | 2 | - | - | - | - | - |
|  | II | - | 41 | - | - | - | - | - | - |
|  | III | 2 | 1 | 9 | - | - | - | - | - |
|  | IV | - | - | - | 6 | - | - | - | - |
| Colony 1 | I | - | - | - | - | 42 | - | 1 | - |
|  | II | 1 | 1 | - | - | 1 | 31 | - | - |
|  | III | - | - | - | - | - | 2 | 20 | - |
|  | IV | - | - | - | - | 1 | - | 2 | 14 |

- 11 Principal Components (46.85% of the shape variation);

95.45% of specimens attributed to their right patriline:

|  |  | Colony 2 | | | | Colony 1 | | | |
| --- | --- | --- | --- | --- | --- | --- | --- | --- | --- |
|  | Patriline | I | II | III | IV | I | II | III | IV |
| Colony 2 | I | 44 | - | 1 | - | - | - | - | - |
|  | II | - | 41 | - | - | - | - | - | - |
|  | III | - | - | 12 | - | - | - | - | - |
|  | IV | - | - | - | 6 | - | - | - | - |
| Colony 1 | I | - | - | - | - | 42 | - | 1 | - |
|  | II | 1 | 1 | - | - | 1 | 31 | - | - |
|  | III | - | - | - | - | - | 2 | 20 | - |
|  | IV | - | - | - | - | 1 | - | 2 | 14 |

- 12 Principal Components (86.92% of the shape variation);

94.55% of specimens attributed to their right patriline:

|  |  | Colony 2 | | | | Colony 1 | | | |
| --- | --- | --- | --- | --- | --- | --- | --- | --- | --- |
|  | Patriline | I | II | III | IV | I | II | III | IV |
| Colony 2 | I | 45 | - | - | - | - | - | - | - |
|  | II | - | 41 | - | - | - | - | - | - |
|  | III | - | - | 12 | - | - | - | - | - |
|  | IV | - | 1 | - | 5 | - | - | - | - |
| Colony 1 | I | - | - | - | - | 40 | 2 | 1 | - |
|  | II | 1 | 1 | - | - | 2 | 30 | - | - |
|  | III | - | - | - | - | - | 2 | 20 | - |
|  | IV | - | - | - | - | 1 | - | 1 | 15 |

- 13 Principal Components (90.56% of the shape variation);

95.45% of specimens attributed to their right patriline:

|  |  | Colony 2 | | | | Colony 1 | | | |
| --- | --- | --- | --- | --- | --- | --- | --- | --- | --- |
|  | Patriline | I | II | III | IV | I | II | III | IV |
| Colony 2 | I | 45 | - | - | - | - | - | - | - |
|  | II | 1 | 40 | - | - | - | - | - | - |
|  | III | - | - | 12 | - | - | - | - | - |
|  | IV | - | - | - | 6 | - | - | - | - |
| Colony 1 | I | - | - | - | - | 42 | - | 1 | - |
|  | II | 1 | 1 | - | - | 2 | 30 | - | - |
|  | III | - | - | - | - | - | 2 | 20 | - |
|  | IV | - | - | - | - | 1 | - | 1 | 15 |

- 14 Principal Components (92.03% of the shape variation);

95.45% of specimens attributed to their right patriline:

|  |  | Colony 2 | | | | Colony 1 | | | |
| --- | --- | --- | --- | --- | --- | --- | --- | --- | --- |
|  | Patriline | I | II | III | IV | I | II | III | IV |
| Colony 2 | I | 45 | - | - | - | - | - | - | - |
|  | II | 1 | 40 | - | - | - | - | - | - |
|  | III | - | - | 12 | - | - | - | - | - |
|  | IV | - | - | - | 6 | - | - | - | - |
| Colony 1 | I | - | - | - | - | 41 | 1 | 1 | - |
|  | II | 1 | 1 | - | - | - | 31 | - | 1 |
|  | III | - | - | - | - | - | 2 | 20 | - |
|  | IV | - | - | - | - | 1 | - | 1 | 15 |

- 15 Principal Components (93.26% of the shape variation);

95.91% of specimens attributed to their right patriline:

|  |  | Colony 2 | | | | Colony 1 | | | |
| --- | --- | --- | --- | --- | --- | --- | --- | --- | --- |
|  | Patriline | I | II | III | IV | I | II | III | IV |
| Colony 2 | I | 45 | - | - | - | - | - | - | - |
|  | II | 1 | 40 | - | - | - | - | - | - |
|  | III | - | - | 12 | - | - | - | - | - |
|  | IV | - | - | - | 6 | - | - | - | - |
| Colony 1 | I | - | - | - | - | 42 | - | 1 | - |
|  | II | - | 1 | - | - | - | 32 | - | 1 |
|  | III | - | - | - | - | 1 | 2 | 19 | - |
|  | IV | - | - | - | - | - | 1 | 1 | 15 |

- 16 Principal Components (94.21% of the shape variation);

96.37% of specimens attributed to their right patriline:

|  |  | Colony 2 | | | | Colony 1 | | | |
| --- | --- | --- | --- | --- | --- | --- | --- | --- | --- |
|  | Patriline | I | II | III | IV | I | II | III | IV |
| Colony 2 | I | 45 | - | - | - | - | - | - | - |
|  | II | 1 | 40 | - | - | - | - | - | - |
|  | III | - | - | 12 | - | - | - | - | - |
|  | IV | - | - | - | 6 | - | - | - | - |
| Colony 1 | I | - | - | - | - | 43 | - | - | - |
|  | II | 1 | 1 | - | - | - | 31 | - | 1 |
|  | III | - | - | - | - | 1 | 2 | 19 | - |
|  | IV | - | - | - | - | - | 1 | - | 16 |

- 17 Principal Components (95.15% of the shape variation);

97.27% of specimens attributed to their right patriline:

|  |  | Colony 2 | | | | Colony 1 | | | |
| --- | --- | --- | --- | --- | --- | --- | --- | --- | --- |
|  | Patriline | I | II | III | IV | I | II | III | IV |
| Colony 2 | I | 45 | - | - | - | - | - | - | - |
|  | II | 1 | 40 | - | - | - | - | - | - |
|  | III | - | - | 12 | - | - | - | - | - |
|  | IV | - | - | - | 6 | - | - | - | - |
| Colony 1 | I | - | - | - | - | 43 | - | - | - |
|  | II | 1 | 1 | - | - | - | 31 | - | 1 |
|  | III | - | - | - | - | - | 1 | 21 | - |
|  | IV | - | - | - | - | - | 1 | - | 16 |

- 18 Principal Components (95.85% of the shape variation);

97.73% of specimens attributed to their right patriline:

|  |  | Colony 2 | | | | Colony 1 | | | |
| --- | --- | --- | --- | --- | --- | --- | --- | --- | --- |
|  | Patriline | I | II | III | IV | I | II | III | IV |
| Colony 2 | I | 45 | - | - | - | - | - | - | - |
|  | II | 1 | 40 | - | - | - | - | - | - |
|  | III | - | - | 12 | - | - | - | - | - |
|  | IV | - | - | - | 6 | - | - | - | - |
| Colony 1 | I | - | - | - | - | 42 | - | 1 | - |
|  | II | - | 1 | - | - | - | 33 | - | - |
|  | III | - | - | - | - | - | 1 | 21 | - |
|  | IV | - | - | - | - | - | 1 | - | 16 |

- 19 Principal Components (96.53% of the shape variation);

99.09% of specimens attributed to their right patriline:

|  |  | Colony 2 | | | | Colony 1 | | | |
| --- | --- | --- | --- | --- | --- | --- | --- | --- | --- |
|  | Patriline | I | II | III | IV | I | II | III | IV |
| Colony 2 | I | 45 | - | - | - | - | - | - | - |
|  | II | 1 | 40 | - | - | - | - | - | - |
|  | III | - | - | 12 | - | - | - | - | - |
|  | IV | - | - | - | 6 | - | - | - | - |
| Colony 1 | I | - | - | - | - | 43 | - | - | - |
|  | II | - | - | - | - | - | 34 | - | - |
|  | III | - | - | - | - | - | 1 | 21 | - |
|  | IV | - | - | - | - | - | - | - | 17 |

- 20 Principal Components (97.13% of the shape variation);

99.09% of specimens attributed to their right patriline:

|  |  | Colony 2 | | | | Colony 1 | | | |
| --- | --- | --- | --- | --- | --- | --- | --- | --- | --- |
|  | Patriline | I | II | III | IV | I | II | III | IV |
| Colony 2 | I | 45 | - | - | - | - | - | - | - |
|  | II | 1 | 40 | - | - | - | - | - | - |
|  | III | - | - | 12 | - | - | - | - | - |
|  | IV | - | - | - | 6 | - | - | - | - |
| Colony 1 | I | - | - | - | - | 43 | - | - | - |
|  | II | - | - | - | - | - | 34 | - | - |
|  | III | - | - | - | - | - | 1 | 21 | - |
|  | IV | - | - | - | - | - | - | - | 17 |

- 21 Principal Components (97.61% of the shape variation);

98.64% of specimens attributed to their right patriline:

|  |  | Colony 2 | | | | Colony 1 | | | |
| --- | --- | --- | --- | --- | --- | --- | --- | --- | --- |
|  | Patriline | I | II | III | IV | I | II | III | IV |
| Colony 2 | I | 45 | - | - | - | - | - | - | - |
|  | II | 1 | 40 | - | - | - | - | - | - |
|  | III | - | - | 12 | - | - | - | - | - |
|  | IV | - | - | - | 6 | - | - | - | - |
| Colony 1 | I | - | - | - | - | 42 | - | - | 1 |
|  | II | - | - | - | - | - | 34 | - | - |
|  | III | - | - | - | - | - | 1 | 21 | - |
|  | IV | - | - | - | - | - | - | - | 17 |

- 22 Principal Components (98.02% of the shape variation);

99.09% of specimens attributed to their right patriline:

|  |  | Colony 2 | | | | Colony 1 | | | |
| --- | --- | --- | --- | --- | --- | --- | --- | --- | --- |
|  | Patriline | I | II | III | IV | I | II | III | IV |
| Colony 2 | I | 45 | - | - | - | - | - | - | - |
|  | II | 1 | 40 | - | - | - | - | - | - |
|  | III | - | - | 12 | - | - | - | - | - |
|  | IV | - | - | - | 6 | - | - | - | - |
| Colony 1 | I | - | - | - | - | 43 | - | - | - |
|  | II | - | - | - | - | - | 34 | - | - |
|  | III | - | - | - | - | - | 1 | 21 | - |
|  | IV | - | - | - | - | - | - | - | 17 |

- 23 Principal Components (98.32% of the shape variation);

98.18% of specimens attributed to their right patriline:

|  |  | Colony 2 | | | | Colony 1 | | | |
| --- | --- | --- | --- | --- | --- | --- | --- | --- | --- |
|  | Patriline | I | II | III | IV | I | II | III | IV |
| Colony 2 | I | 45 | - | - | - | - | - | - | - |
|  | II | 1 | 39 | 1 | - | - | - | - | - |
|  | III | - | - | 12 | - | - | - | - | - |
|  | IV | - | - | - | 6 | - | - | - | - |
| Colony 1 | I | - | - | - | - | 43 | - | - | - |
|  | II | - | - | - | - | - | 34 | - | - |
|  | III | - | - | - | - | - | 1 | 21 | - |
|  | IV | - | - | - | - | - | - | 1 | 16 |

- 24 Principal Components (98.59% of the shape variation);

97.73% of specimens attributed to their right patriline:

|  |  | Colony 2 | | | | Colony 1 | | | |
| --- | --- | --- | --- | --- | --- | --- | --- | --- | --- |
|  | Patriline | I | II | III | IV | I | II | III | IV |
| Colony 2 | I | 45 | - | - | - | - | - | - | - |
|  | II | 1 | 39 | 1 | - | - | - | - | - |
|  | III | - | - | 12 | - | - | - | - | - |
|  | IV | - | - | - | 6 | - | - | - | - |
| Colony 1 | I | - | - | - | - | 42 | 1 | - | - |
|  | II | - | - | - | - | - | 34 | - | - |
|  | III | - | - | - | - | - | 1 | 21 | - |
|  | IV | - | - | - | - | - | - | 1 | 16 |

- 25 Principal Components (98.8% of the shape variation);

97.27% of specimens attributed to their right patriline:

|  |  | Colony 2 | | | | Colony 1 | | | |
| --- | --- | --- | --- | --- | --- | --- | --- | --- | --- |
|  | Patriline | I | II | III | IV | I | II | III | IV |
| Colony 2 | I | 45 | - | - | - | - | - | - | - |
|  | II | 1 | 39 | 1 | - | - | - | - | - |
|  | III | - | - | 12 | - | - | - | - | - |
|  | IV | - | - | - | 6 | - | - | - | - |
| Colony 1 | I | - | - | - | - | 41 | 1 | - | 1 |
|  | II | - | - | - | - | - | 34 | - | - |
|  | III | - | - | - | - | - | 1 | 21 | - |
|  | IV | - | - | - | - | 1 | - | - | 16 |

- 26 Principal Components (99.01% of the shape variation);

97.27% of specimens attributed to their right patriline:

|  |  | Colony 2 | | | | Colony 1 | | | |
| --- | --- | --- | --- | --- | --- | --- | --- | --- | --- |
|  | Patriline | I | II | III | IV | I | II | III | IV |
| Colony 2 | I | 45 | - | - | - | - | - | - | - |
|  | II | 1 | 39 | 1 | - | - | - | - | - |
|  | III | - | - | 12 | - | - | - | - | - |
|  | IV | - | - | - | 6 | - | - | - | - |
| Colony 1 | I | - | - | - | - | 41 | 1 | - | 1 |
|  | II | - | - | - | - | - | 34 | - | - |
|  | III | - | - | - | - | - | 2 | 20 | - |
|  | IV | - | - | - | - | - | - | - | 17 |

- 27 Principal Components (99.2% of the shape variation);

97.27% of specimens attributed to their right patriline:

|  |  | Colony 2 | | | | Colony 1 | | | |
| --- | --- | --- | --- | --- | --- | --- | --- | --- | --- |
|  | Patriline | I | II | III | IV | I | II | III | IV |
| Colony 2 | I | 45 | - | - | - | - | - | - | - |
|  | II | 1 | 40 | - | - | - | - | - | - |
|  | III | - | - | 12 | - | - | - | - | - |
|  | IV | - | - | - | 6 | - | - | - | - |
| Colony 1 | I | - | - | - | - | 41 | 1 | - | 1 |
|  | II | - | - | - | - | - | 34 | - | - |
|  | III | - | - | - | - | - | 2 | 20 | - |
|  | IV | - | - | - | - | - | - | 1 | 16 |

- 28 Principal Components (99.38% of the shape variation);

97.73% of specimens attributed to their right patriline:

|  |  | Colony 2 | | | | Colony 1 | | | |
| --- | --- | --- | --- | --- | --- | --- | --- | --- | --- |
|  | Patriline | I | II | III | IV | I | II | III | IV |
| Colony 2 | I | 45 | - | - | - | - | - | - | - |
|  | II | - | 41 | - | - | - | - | - | - |
|  | III | - | - | 12 | - | - | - | - | - |
|  | IV | - | - | - | 6 | - | - | - | - |
| Colony 1 | I | - | - | - | - | 42 | - | - | 1 |
|  | II | - | - | - | - | 1 | 33 | - | - |
|  | III | - | - | - | - | - | 2 | 20 | - |
|  | IV | - | - | - | - | - | - | 1 | 16 |

- 29 Principal Components (99.54% of the shape variation);

96.82% of specimens attributed to their right patriline:

|  |  | Colony 2 | | | | Colony 1 | | | |
| --- | --- | --- | --- | --- | --- | --- | --- | --- | --- |
|  | Patriline | I | II | III | IV | I | II | III | IV |
| Colony 2 | I | 45 | - | - | - | - | - | - | - |
|  | II | - | 40 | 1 | - | - | - | - | - |
|  | III | - | - | 12 | - | - | - | - | - |
|  | IV | - | - | - | 6 | - | - | - | - |
| Colony 1 | I | - | - | - | - | 41 | - | 1 | 1 |
|  | II | - | - | - | - | - | 34 | - | - |
|  | III | - | - | - | - | - | 2 | 20 | - |
|  | IV | - | - | - | - | 1 | - | 1 | 15 |

- 30 Principal Components (99.66% of the shape variation);

97.27% of specimens attributed to their right patriline:

|  |  | Colony 2 | | | | Colony 1 | | | |
| --- | --- | --- | --- | --- | --- | --- | --- | --- | --- |
|  | Patriline | I | II | III | IV | I | II | III | IV |
| Colony 2 | I | 45 | - | - | - | - | - | - | - |
|  | II | - | 40 | 1 | - | - | - | - | - |
|  | III | - | - | 12 | - | - | - | - | - |
|  | IV | - | - | - | 6 | - | - | - | - |
| Colony 1 | I | - | - | - | - | 42 | - | - | 1 |
|  | II | - | - | - | - | 1 | 33 | - | - |
|  | III | - | - | - | - | - | 2 | 20 | - |
|  | IV | - | - | - | - | 1 | - | - | 16 |

- 31 Principal Components (99.78% of the shape variation);

97.27% of specimens attributed to their right patriline:

|  |  | Colony 2 | | | | Colony 1 | | | |
| --- | --- | --- | --- | --- | --- | --- | --- | --- | --- |
|  | Patriline | I | II | III | IV | I | II | III | IV |
| Colony 2 | I | 45 | - | - | - | - | - | - | - |
|  | II | - | 40 | 1 | - | - | - | - | - |
|  | III | - | - | 12 | - | - | - | - | - |
|  | IV | - | - | - | 6 | - | - | - | - |
| Colony 1 | I | - | - | - | - | 42 | - | - | 1 |
|  | II | - | - | - | - | 1 | 33 | - | - |
|  | III | - | - | - | - | - | 2 | 20 | - |
|  | IV | - | - | - | - | - | - | 1 | 16 |

- 32 Principal Components (99.86% of the shape variation);

97.27% of specimens attributed to their right patriline:

|  |  | Colony 2 | | | | Colony 1 | | | |
| --- | --- | --- | --- | --- | --- | --- | --- | --- | --- |
|  | Patriline | I | II | III | IV | I | II | III | IV |
| Colony 2 | I | 45 | - | - | - | - | - | - | - |
|  | II | - | 40 | 1 | - | - | - | - | - |
|  | III | - | - | 12 | - | - | - | - | - |
|  | IV | - | - | - | 6 | - | - | - | - |
| Colony 1 | I | - | - | - | - | 42 | - | - | 1 |
|  | II | - | - | - | - | 1 | 33 | - | - |
|  | III | - | - | - | - | - | 2 | 20 | - |
|  | IV | - | - | - | - | - | - | 1 | 16 |

- 33 Principal Components (99.94% of the shape variation);

97.27% of specimens attributed to their right patriline:

|  |  | Colony 2 | | | | Colony 1 | | | |
| --- | --- | --- | --- | --- | --- | --- | --- | --- | --- |
|  | Patriline | I | II | III | IV | I | II | III | IV |
| Colony 2 | I | 45 | - | - | - | - | - | - | - |
|  | II | - | 40 | 1 | - | - | - | - | - |
|  | III | - | - | 12 | - | - | - | - | - |
|  | IV | - | - | - | 6 | - | - | - | - |
| Colony 1 | I | - | - | - | - | 42 | - | - | 1 |
|  | II | - | - | - | - | 1 | 33 | - | - |
|  | III | - | - | - | - | 1 | 1 | 20 | - |
|  | IV | - | - | - | - | - | - | 1 | 16 |

- 34 Principal Components (100% of the shape variation);

97.27% of specimens attributed to their right patriline:

|  |  | Colony 2 | | | | Colony 1 | | | |
| --- | --- | --- | --- | --- | --- | --- | --- | --- | --- |
|  | Patriline | I | II | III | IV | I | II | III | IV |
| Colony 2 | I | 45 | - | - | - | - | - | - | - |
|  | II | 1 | 40 | - | - | - | - | - | - |
|  | III | - | - | 12 | - | - | - | - | - |
|  | IV | - | - | - | 6 | - | - | - | - |
| Colony 1 | I | - | - | - | - | 42 | - | - | 1 |
|  | II | - | - | - | - | 1 | 33 | - | - |
|  | III | - | - | - | - | 1 | 1 | 20 | - |
|  | IV | - | - | - | - | - | - | 1 | 16 |
